# Editorial: Chemical and biological changes of polyphenols caused by food thermal processing

**DOI:** 10.3389/fnut.2022.948894

**Published:** 2022-07-29

**Authors:** Ying Zhang, Huilin Liu

**Affiliations:** Beijing Technology and Business University, Beijing, China

**Keywords:** polyphenols, Maillard reaction, high temperature, food thermal processing, advanced glycation endproducts

The plant polyphenols found in foods are phenolic secondary metabolites with natural bioactivity and can be considered a key element in food processing. Polyphenols can not only directly enrich the nutritional value of food, but also affect the quality of the final product as functional factors (1). Polyphenols are commonly classified as flavonoids and non-flavonoids (including phenolic acids, lignans, and stilbenes) based on their chemical structure. Thermal processing is an important processing method for food that can enhance nutritional value, ensuring it can be safely consumed, maintaining organoleptic properties, and extending the shelf life of products. Due to the considerable thermos-reactivity and sensitivity of polyphenols, the modification of different reaction pathways during thermal processing has led to significant chemical and biological changes in polyphenols (2).

This Research Topic focuses on improving our knowledge and understanding of how thermal processing affects polyphenols, and both the positive and negative effects of any chemical and biological changes.

Different thermal processing technologies can affect polyphenols in foods in different ways, resulting in different effects on food-borne polyphenols. Jan et al. reported that during apricot canning and storage, chemical oxidation at high temperatures caused the polymerization and degradation of polyphenols, resulting in a decrease in total phenolic content. Martini et al. showed that frying significantly increased the main polyphenols, hydroxycinnamic acids, in dark purple eggplant, because of both isomerization and hydrolysis reactions, whereas negative effects were produced by boiling, grilling, and baking (3). These results indicated that the chemical properties of polyphenols were changed during thermal processing through epimerization, oxidative polymerization, and degradation. These changes were accompanied by biological activity associated with increasing or decreasing the polyphenol content.

A growing body of literature has investigated the chemical and biological effects of thermal processing on polyphenol-mediated processes in food systems. Polyphenols can directly interact with intermediates during thermal processing (i.e., carbonyl-containing compounds) through Maillard reactions, lipid oxidation, and sugar condensation pathways. Of the various processes associated with Maillard reactions, polyphenol-mediated thermal processes can be beneficial by reacting with the active intermediates of advanced glycation endproducts (AGEs) (such as glyoxal, methylglyoxal, and 3-deoxyglucosone) to form the corresponding carbonyl-phenol adducts (4). Zhang et al. reported that nine major flavonoids from corn silk reacted with glyoxal and methylglyoxal to form mono-, di-, and tri-adducts in a casein glucose-fatty acid model system (treated at 120°C for 30 min), which inhibited the formation of exogenous AGEs (*N*^ε^-carboxymethyllysine). Thermal processing can also change the chemical properties and structures of polyphenols to positively influence food processing (5). Another study performed by Poojary et al. also found that after epigallocatechin gallate (EGCG) in milk was epimerizatized into gallocatechin gallate (GCG) during the ultrahigh temperature processing, EGCG/GCG-based α-dicarbonyl adducts and hydroxymethylfurfural adducts were formed. However, lysine-derived AGEs [*N*^ε^-carboxymethyllysine and N^ε^-(carboxyethyl)lysine] accumulated abnormally during this process, indicating that ultrahigh temperature processing did not necessarily exert positive chemical and biological effects on polyphenol-mediated processes (6).

Polyphenols, through chemical changes, have also been shown to have inhibitory effects on acrylamide, another common thermally induced chemical contaminant. Mildner-Szkudlarza et al. proposed that ferulic acid in their model wheat bread system inhibited the generation of undesirable acrylamide derived from the Maillard reaction. The inhibition mechanism was assigned to the direct trapping of acrylamide precursors by polyphenols, but the specific inhibition mechanism and pathway needs further researched (7). In addition, the interaction between lipid-derived carbonyl structures and polyphenols was also an important consideration. Data from Hidalgo et al. showed that the addition reaction of polyphenols with the secondary lipid oxidation products of 4-hydroxy-2-nonenal (three adducts) and 4-hydroxy-2-hexenal (one adduct) during thermal processing at 80°C contributed to the inhibition of lipid oxidation (8). Overall, thermal processing can induce chemical and biological changes in polyphenols, involving direct interactions mediated by polyphenol with intermediates related to thermal processing, that can play a positive or a negative role in food processing.

In contrast to these processes mediated by polyphenol, polyphenols and their oxide quinones could bind with nucleophilic residues on proteinaceous components through covalent and non-covalent bonds during thermal processing. The binding of polyphenols to proteins can enhance resistance to the thermal degradation of polyphenols. The quality of milk is always affected by lipid oxidation, particularly after heat processing. Natural active polyphenols are commonly considered to be thermally sensitive and effective antioxidants. Tian et al. showed that anthocyanins were stabilized during thermal processing through their interaction with casein. The formed anthocyanin-casein complex could promote iron auto-oxidation and inhibit lipid peroxidation, which was verified in the purple corn anthocyanin-supplemented milk system with the potential to prevent milk oxidation after pasteurization. Outside of the direct thermo-sensitivity, anthocyanin could also be significantly more affected under blue light irradiation compared with irradiation from other visible light spectra (red, green, and white), which was confirmed by the transcriptomic and metabolomic profiling in the research of Zhang et al. The chemical structure and biological activity of polyphenols also depended on the interaction with polysaccharides. Li et al. verified the chemical conjugation of polyphenols (catechin and gallic acid) with polysaccharides that were thermally induced at 40°C in lotus root, which increased the thermal stability of the complexes and the biological activities of polyphenols and monosaccharides (9). Using the same method at 30°C, Yi et al. found that polyphenols including (catechin, gallic acid, ferric acid, chlorogenic acid, and caffeic acid) interacted with lotus root polysaccharides through non-covalent intermolecular interaction. This provided experimental evidence that thermal processing can be used to alter the interaction between polyphenols and polysaccharides (10).

Growing awareness of the health benefits associated with polyphenols has increased the requirement for preserving their nutritional value as well as exerting greater biological function. The research presented in this review provides a mini-overview of changes in the chemical and biological aspects of polyphenols during thermal processing, focusing on the properties of polyphenols, polyphenol-mediated processes, and their interactions with other components. Different pathways in thermal processing decreased the content of polyphenols, but in some cases increased it. Furthermore, the trapping reaction in polyphenol-mediated processing generated polyphenol-based adducts and the binding of polyphenols with macromolecular components are both crucial for understanding the chemical or biological effects of thermal processing on polyphenols. This paper indicates that thermal processing is of great significance to foodborne polyphenols and provides useful information for further explaining the chemical and biological changes of polyphenols in response to thermal processing.

## Author contributions

HL proposed the scope and concept of the present Research Topic and wrote the introduction and the conclusion. YZ prepared the main part with comments on the cited papers and references, which were reviewed by HL. All authors contributed to the article and approved the submitted version.

## Funding

This work was supported by the National Natural Science Foundation of China (Nos. 32072335 and 31822040).

## Conflict of interest

The authors declare that the research was conducted in the absence of any commercial or financial relationships that could be construed as a potential conflict of interest.

## Publisher's note

All claims expressed in this article are solely those of the authors and do not necessarily represent those of their affiliated organizations, or those of the publisher, the editors and the reviewers. Any product that may be evaluated in this article, or claim that may be made by its manufacturer, is not guaranteed or endorsed by the publisher.
